# The metabolomic signature of extreme longevity: naked mole rats *versus* mice

**DOI:** 10.18632/aging.102116

**Published:** 2019-07-24

**Authors:** Mélanie Viltard, Sylvère Durand, Maria Pérez-Lanzón, Fanny Aprahamian, Deborah Lefevre, Christine Leroy, Frank Madeo, Guido Kroemer, Gérard Friedlander

**Affiliations:** 1 Fondation pour la Recherche en Physiologie, Brussels, Belgium; 2 Metabolomics and Cell Biology Platforms, Institut Gustave Roussy, Villejuif, France; 3 Equipe Labellisée par la Ligue contre le Cancer, Université de Paris, Sorbonne Université, INSERM U1138, Centre de Recherche des Cordeliers, Paris, France; 4 Faculté de Médecine, Université de Paris Saclay, Kremlin Bicêtre, France; 5 INSERM UMR_S1151 CNRS UMR8253 Institut Necker-Enfants Malades (INEM), Paris, France; 6 Institute of Molecular Biosciences, University of Graz, NAWI Graz, Graz, Austria; 7 BioTechMed Graz, Graz, Austria; 8 Pôle de Biologie, Hôpital Européen Georges Pompidou, AP-HP, Paris, France; 9 Suzhou Institute for Systems Medicine, Chinese Academy of Sciences, Suzhou, China; 10 Karolinska Institute, Department of Women's and Children's Health, Karolinska University Hospital, Stockholm, Sweden; 11 Service de Physiologie et Explorations Fonctionnelles, Hôpital Européen Georges Pompidou, Assistance Publique-Hôpitaux de Paris, Paris, France; 12 Université de Paris - Paris Descartes, Faculté de Médecine, Paris, France

**Keywords:** spermidine, antioxidants, autophagy, catabolism, meta-organism, microbiota

## Abstract

The naked mole-rat (*Heterocephalus glaber*) is characterized by a more than tenfold higher life expectancy compared to another rodent species of the same size, namely, the laboratory mouse (*Mus musculus*). We used mass spectrometric metabolomics to analyze circulating plasma metabolites in both species at different ages. Interspecies differences were much more pronounced than age-associated alterations in the metabolome. Such interspecies divergences affected multiple metabolic pathways involving amino, bile and fatty acids as well as monosaccharides and nucleotides. The most intriguing metabolites were those that had previously been linked to pro-health and antiaging effects in mice and that were significantly increased in the long-lived rodent compared to its short-lived counterpart. This pattern applies to α-tocopherol (also known as vitamin E) and polyamines (in particular cadaverine, N8-acetylspermidine and N1,N8-diacetylspermidine), all of which were more abundant in naked mole-rats than in mice. Moreover, the age-associated decline in spermidine and N1-acetylspermidine levels observed in mice did not occur, or is even reversed (in the case of N1-acetylspermidine) in naked mole-rats. In short, the present metabolomics analysis provides a series of testable hypotheses to explain the exceptional longevity of naked mole-rats.

## Introduction

Although biological and chronological time can be dissociated to some extent by experimental manipulation, aging appears to be the most important risk factor for the deterioration of normal physiological functions and the manifestation of organ-specific or systemic pathologies, physical and mental decadency, and eventual death [1–3].

One species that – to a certain degree – escapes from the rule that natural life expectancy declines with body mass is the naked mole-rat (*Heterocephalus glaber*). The naked mole-rat (NMR) is a small poikilotherm rodent native to East Africa that lives strictly underground in social colonies (Kenya, Ethiopia and Somalia). Although this rodent has a similar size as the laboratory mouse (*Mus musculus*), it lives 10-20 times longer without showing any visible signs of aging [4]. Furthermore, the naked mole-rat can live for over 32 years in captivity [5], without facing any increased age-related risk of mortality, challenging Gompertz’s mortality law, and thus establishing the naked mole-rat as a non-aging mammal [6].

Not only naked mole-rats can live an extremely long life, but they also show a remarkably long healthspan associated with almost no decline in physiological or biochemical functions for more than 20 years [4,7]. For example, cardiac functions are well preserved in aged naked mole-rats [8], cognitive functions do not decline with age and the NMR brain seems to be naturally protected from neurodegenerative processes [9], and also very little pathologic alterations have been found in the kidneys of aged naked mole-rats [10].

In addition, typical signs of aging, such as loss of fertility, muscle atrophy, bone loss, changes in body composition or metabolism are mostly absent in the naked mole-rats [7,11–13]. Finally, the incidence of age-related diseases such as cancers or metabolic disorders is extremely low in the NMR [10,14].

Herein, we investigated age-dependent and species-specific differences in the metabolome of naked mole-rats and mice, with the objective to identify novel mechanisms that may explain the exceptional resistance of NMR against the advancement of time. We were able to identify several circulating metabolites previously associated to an increased healthspan and lifespan in other species that might explain the longevity phenotype of this model species.

## RESULTS AND DISCUSSION

### Trans-species differences in the metabolome

**Figure 1:**
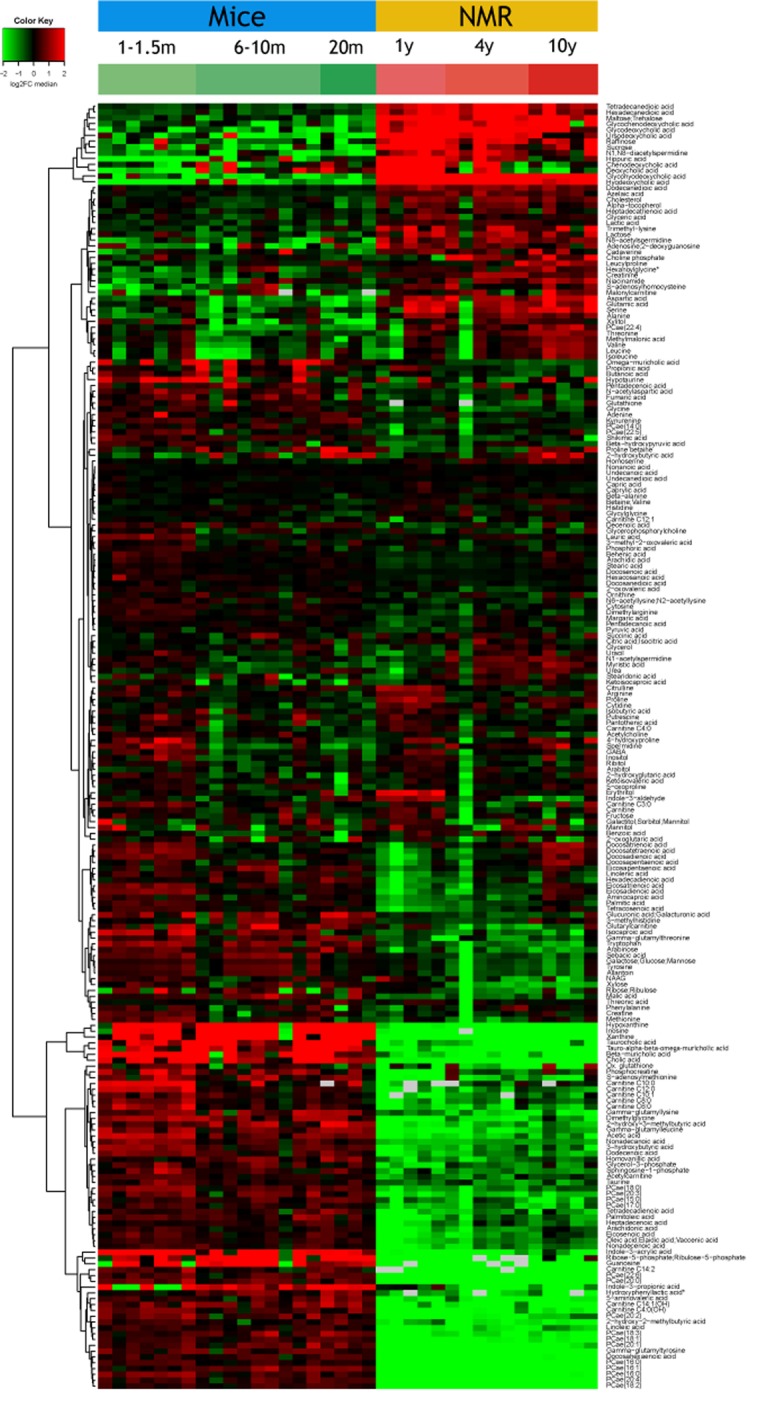
**Overview of the plasma metabolome in the two rodents.** The abundance of each metabolite is indicated for each mouse or naked mole-rat (NMR) as a heat map (red = high, green=low). Results were subjected to hierarchical clustering to indicate the increase (upper part) or decreased (lower part) of metabolites in NMR as compared to mice. Note that the raw data are listed in Supplementary Table 1.

**Figure 2:**
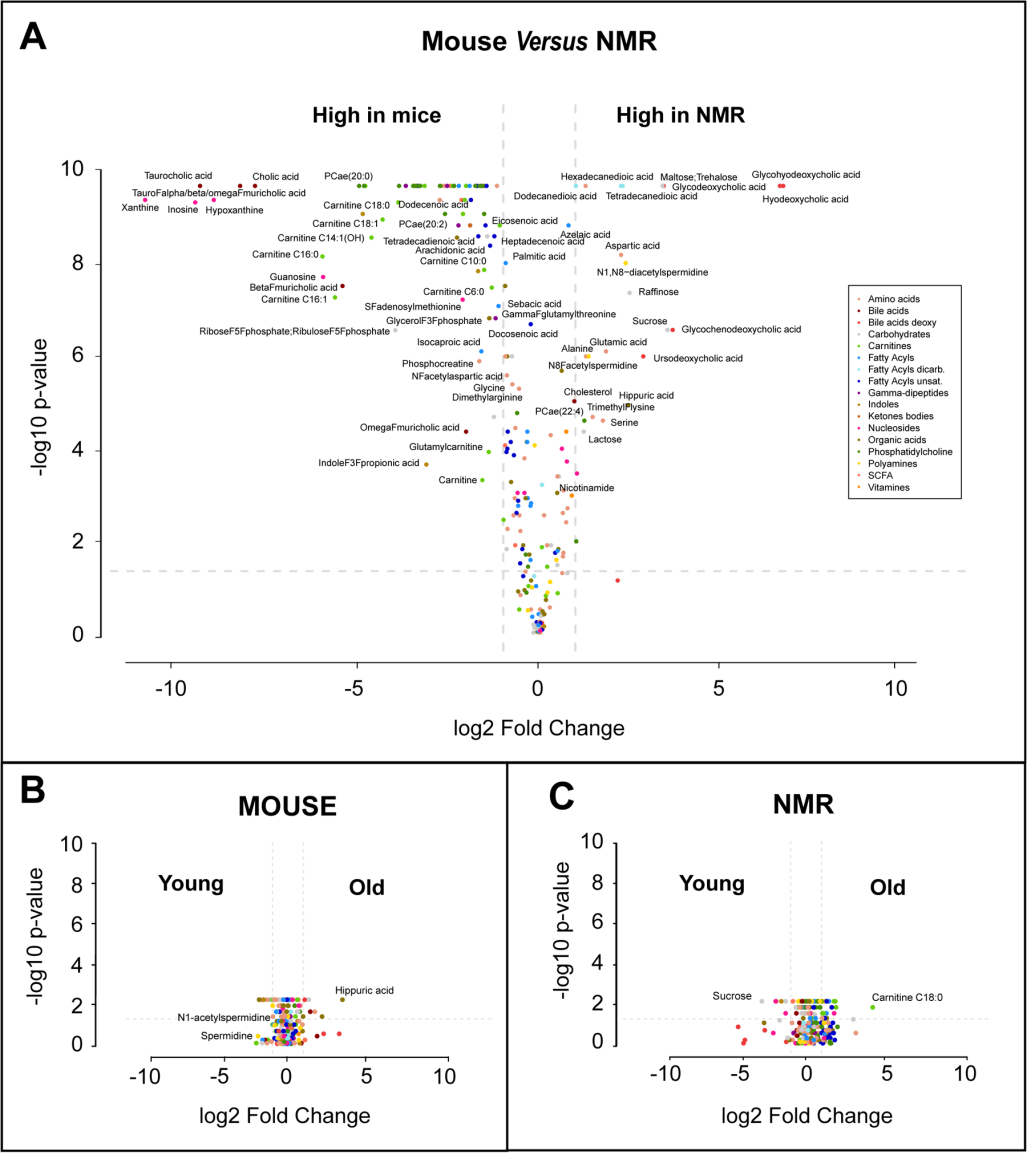
**Volcano plots of metabolome differences.** (**A**) Interspecies comparison. (**B**) Comparison between young (1-1.5 months) and old (20 months) mice. (**C**) Comparison between young (1 year) and old (10 months) naked mole-rats (NMR). The color code classifying different metabolic species used in **A** is also used in **B** and **C**. Selected metabolites are indicated.

Plasma samples from post-adolescent young (1-1.5 months), intermediate (6-10 months) and mature/old (20 months) mice were compared to plasma specimens from post-adolescent young (1 year), intermediate (4 years) and relatively mature/old (10 years) naked mole-rats. Samples were subjected to mass spectrometric metabolomics using several different extraction methods, matrices and chromatography methods (including gas and liquid chromatography) to extract a maximum of information on a wide spectrum of metabolites. Results were then filtered based on quality control criteria (see Materials and Methods) and subjected to unbiased hierarchical clustering to reveal age- and species-dependent differences (Figure 1, Supplementary Table 1). We also performed volcano plot-based comparisons between mice and naked mole-rats irrespective of their age (Figure 2a), as well as within the same species between young and old animals (Figure 2a, b). This approach clearly indicates that species differences are well more important than age, allowing to clearly separating the samples from two rodents (Figure 1). The number of metabolites that were significantly reduced in their plasma concentration in naked mole-rats as compared to mice was larger than the number of compounds that were enhanced (Figure 1 and 2, Supplementary Table 1). Bioinformatic analyses to understand the divergence in the metabolomes from mice and naked mole-rats failed to yield a simple pattern of differences (Supplementary Figure 1 and Supplementary Figure 2). Rather, the two species differ in multiple apparently unconnected pathways.

### Metabolites reduced in naked mole-rats

A large number of diverse circulating phosphatidyl cholines (abbreviated PCae) with distinct acyl chains (length 14 to 20) and unsaturation levels (from 0 to 4) were reduced in naked mole-rats. In addition, multiple unsaturated free fatty acids (e.g. arachidonic, dodecenoic, docosahexaenoic, heptadecanoic, linoleic, nonadecanoic, oleic, palimitoleic, tetradecadienoic acids) and carnitine-acyl esters (length 2-14) were decreased, pointing to a major alteration of lipid metabolism. The reduction of lipids and in particular, carnitine-acyl esters, that occurs in naked mole-rats might indicate efficient beta-oxidation. In line with a possible alteration of lipid metabolism, the major ketone body 3-hydroxybutyrate and two ketogenesis-associated metabolites, 2-hydroxy-2-methylbutyric acid and 2-hydroxy-3-methylbutyric acid, were depleted in the long-lived species. Furthermore, two primary bile acids (cholic and muricholic acids) and two tauro-conjugated bile acids (taurocholic, tauromuricholic acids) were strongly diminished. Several nucleic acid-relevant metabolites (guanosine, hypoxanthine, inosine, ribose-5-phosphate, and xanthine) were also reduced in the plasma from naked mole-rats compared to that of mice (Figure 1 and Figure 2a).

**Figure 3:**
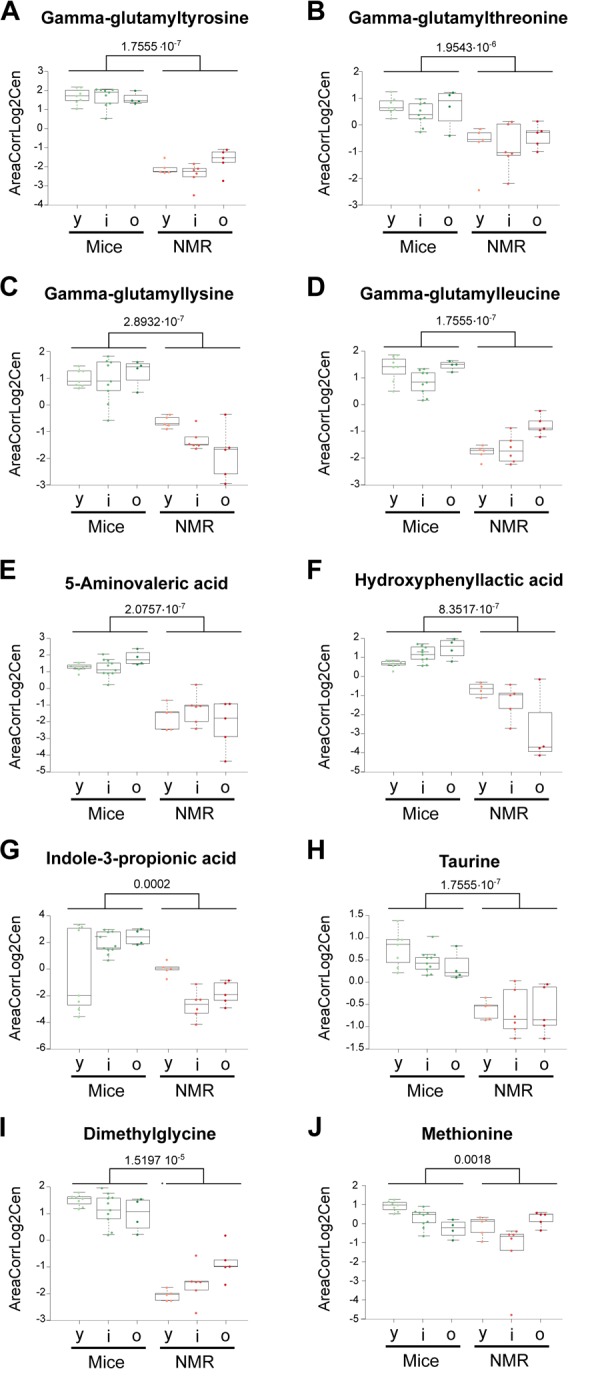
**Amino acid derivatives that are reduced in naked mole-rats.** Statistical comparisons were calculated by means of a two-sided Wilcoxon test. P-values are indicated.

Several gamma-glutamyl amino acids (gamma-glutamyl leucine, gamma-glutamyl lysine, gamma-glutamyl threonine, and gamma-glutamyl tyrosine), which are proteolytic breakdown products were underrepresented in the plasma from naked mole-rats, in line with a prior report [15], suggesting a major reduction in protein turnover or an improved clearance of these metabolites (Figure 3a-d). The lysine degradation product 5-aminovaleric acid present also a reduced abundance in NMR compared to mice (Figure 3e). This metabolite is correlated positively with breast cancer risk in women [16]. Hydroxyphenyllactic acid, a tyrosine metabolite that increases with weight loss in obese women [17] and that correlates with ovarian cancer recurrence after surgery [18], was also decreased in NMR (Figure 3f). Furthermore, the tryptophan metabolite 3-Indole propionic acid (a bacterial metabolite), was diminished (Figure 3g). The same applies to taurine, a cysteine metabolite (Figure 3h), and dimethylglycine (Figure 3i), which increases with methionine restriction in mice [19]. However, methionine tended to be relatively lower in the long-lived species (Figure 3j), supporting the hypothesis [15] that long lived naked mole-rats bear characteristics of a methionine-restricted metabolism.

**Figure 4:**
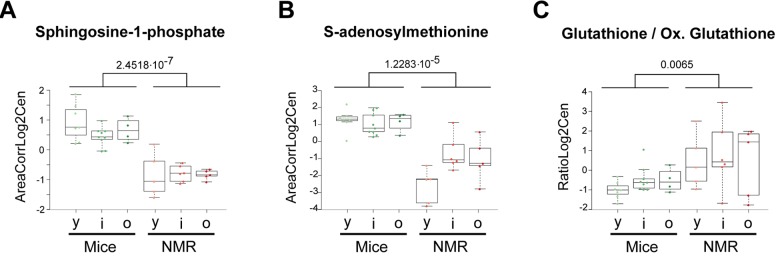
**Selected metabolic alterations in naked mole-rats.** Statistical comparisons were calculated by means of a two-sided Wilcoxon test. P-values are indicated.

Of note, sphingosine-1-phosphates (S1P) was reduced in naked mole-rats compared to mice (Figure 4a), a finding that appears interesting because in humans, S1P levels inversely associate with arteriosclerosis [20], but increase in prostate cancer [21], and predict osteoporotic fractures in postmenopausal women [22]. Thus, S1P seems a biomarker of several health-related parameters. Other metabolites that were reduced in the long-living species were glycerol-3-phosphate, indole-3-acrylic acid (a plant auxin), phosphocreatine (Figure 1), S-adenosylmethione (the methyl group donor for methylation reactions) (Figure 4b), as well oxidized glutathione, pleading in favor of a strong antioxidant system [23]. Indeed, the ratio of reduced over oxidized glutathione was significantly higher in naked mole-rats than in mice (Figure 4c).

Thus, a large panel of metabolites falling into distinct functional categories is comparatively low-abundant in naked mole-rats than in mice.

### Metabolites increased in naked mole-rats

Opposite to the multiple abovementioned phosphatidyl cholines, PCae (22:4) was increased in naked mole-rats. Three aliphatic even-number medium-chain (12, 14 or 16 carbons) alpha-omega dicarboxylic acids (dodecanedioic, tetradecanedioic and heptadecatrienoic acids) were increased in NMR. These metabolites can be endogenously generated by omega oxidation of monocarboxylic acids or stem from vegetables [24]. Among these, dodecanedioic acid has been shown to improve glycemic control and to reduce muscle fatigue in type-2 diabetes patients, suggesting that it can improve metabolic flexibility [25,26]. In contrast, hexadecanedioic acid plasma levels are associated with high blood pressure in patients, and oral supplementation of hexadecanedioic acid causes hypertension in rats [27] (Figure 1).

Phosphocholine and cholesterol were enhanced in the long-lived rodent (Figure 1). Several bile acids were also increased, as this applies to one primary bile acid (chenodeoxycholic), three secondary bile acids (deoxycholic, hyodeoxycholic, ursodeoxycholic) and three glycoconjugated (glycodeoxycholic, glycochenodeoxycholic, glycohyodeoxycholic. This shift in circulating bile acids (Supplementary Figure 1) suggests species-differences in their metabolism, as this has been reported across mammalian species [28]. Bile acid supplementation has been shown to promote longevity in yeast [29,30], and supplementation with cholic acid increases longevity in short-lived progeroid mice [19]. This suggests a possible link between bile acid metabolism and the exceptional longevity of naked mole-rats.

**Figure 5:**
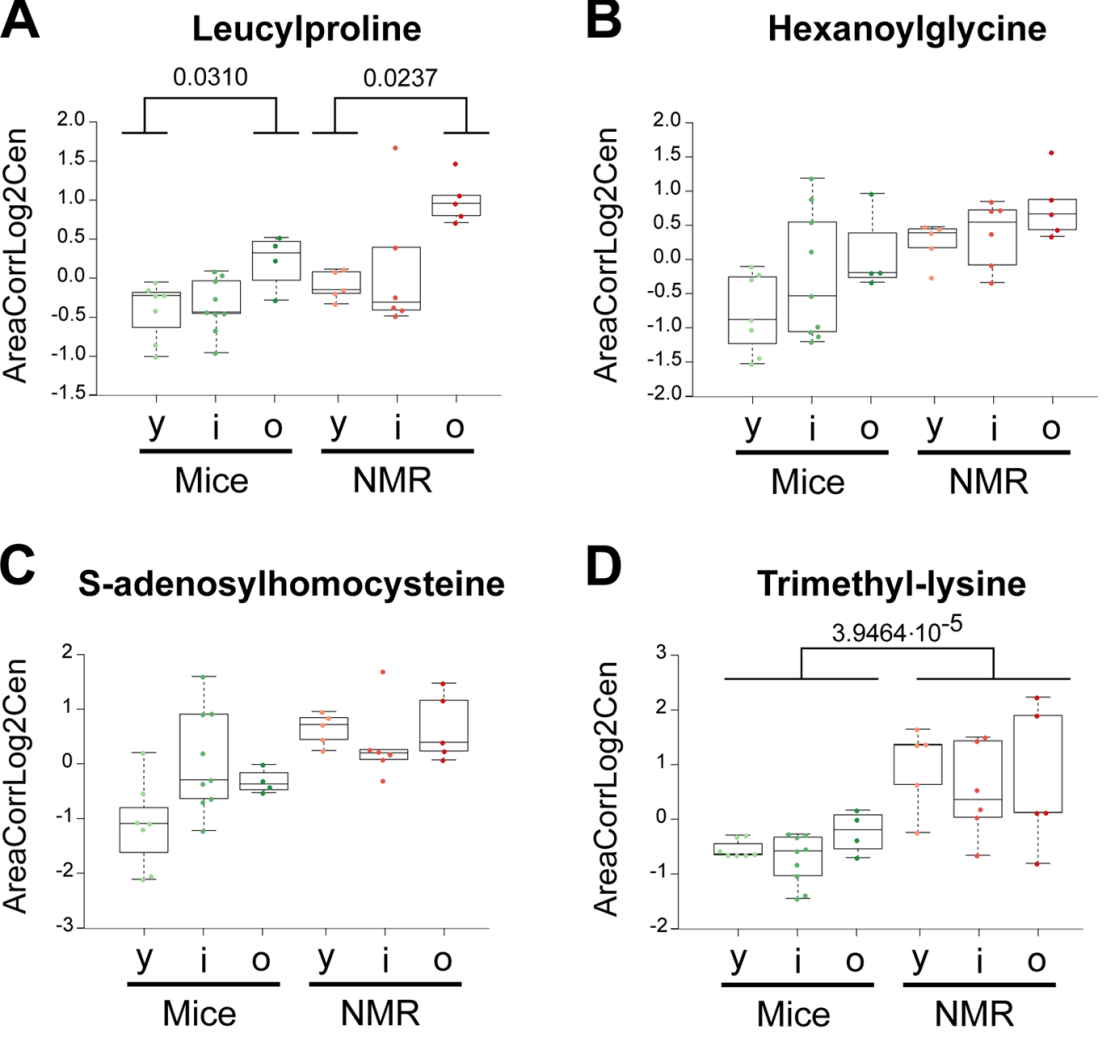
**Amino acid derivatives that are elevated in naked mole-rats.** Statistical comparisons were calculated by means of a two-sided Wilcoxon test. P-values are indicated.

Several free amino acids were particularly abundant in naked mole-rats compared to mice: alanine, aspartic acid, glutamic acid, isoleucine, leucine, serine and threonine (Figure 1, Figure 2a). Moreover, the dipeptide leucylproline and several amino acid derivatives were elevated: hexanoylglycine, S-adenosyl homocysteine and trimethyl lysine (Figure 5a-d). Interestingly, multiple sugars were more abundant in the plasma from the long-lived species, as this applies to lactose, maltose, raffinose and sucrose, as well as to the sugar alcohol xylitol. Two malonate derivatives, methylmalonic acid and malonylcarnitine were both overabundant in naked mole-rats. Lactate was increased, and so was adenosine, azelaic acid (generally considered as a fungal metabolite), creatinine, glyceric acid (a glycerol oxidation product) and hippuric acid (a biomarker of polyphenol uptake) (Figure 1, Figure 2a).

**Figure 6:**
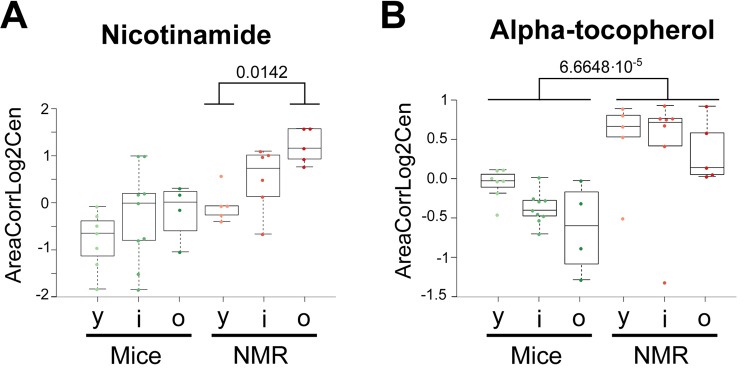
**Vitamins that are elevated in naked mole-rats.** Statistical comparisons were calculated by means of a two-sided Wilcoxon test. P-values are indicated.

**Figure 7:**
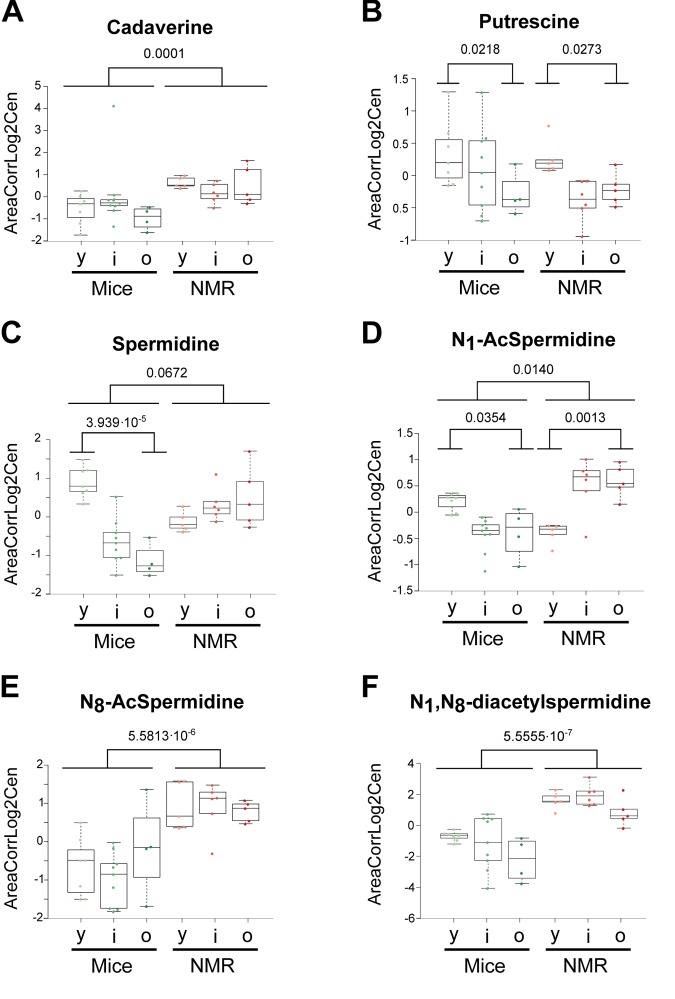
**Influence of age and species differences on the abundance of polyamines and polyamine metabolites.** Statistical comparisons were calculated by means of a two-sided Wilcoxon test. P-values are indicated.

The few metabolites that have been most convincingly linked to health-improving and antiaging effects in mice include two vitamins (B3, E) and polyamines. Nicotinamide (also called niacinamide, vitamin B3), which is known to extend health span and lifespan in mice [31,32], tend to increase in naked mole-rats and actually augmented with age in this species but not in mice (Figure 6a). The potent antioxidant α-tocopherol (vitamin E), which can enhance the lifespan of short-lived mouse strains [33,34], was also increased (Figure 6b). Several polyamines (cadaverine, N8-acetylspermidine, N1,N8-diacetylspermidine, but not ornithine nor putrescine) were elevated in the long-lived species (Figure 7). Importantly, spermidine and N1 acetylspermidine declined in aging mice, but remained at high levels in aging naked mole-rats (Figure 7c, d), echoing an abundant literature showing that spermidine supplementation promotes longevity in mice and other model organisms [35–38] and that increased nutrient uptake of spermidine reduces cardiovascular and cancer-related mortality in humans [39–41].

## ConcluSIONS

Our present work reveals important differences in the metabolism between two species differing in their natural lifespan, namely, short-lived mice and long-lived naked mole-rats. These differences affect all major metabolic pathways, leading to alterations in the relative proportions of specific amino acid and their derivatives, free fatty acids and their carnitine esters, phosphatidyl cholines, bile acids, nucleic acids and their derivatives, protein degradation products and many other metabolites. A few differences may be hypothesis generating, as this applies to nicotinamide, α-tocopherol and polyamines which augmented in naked mole-rats, in the line with prior experiments showing that their continuous administration to mice can increase health span and longevity. That said, it will be necessary to inhibit the pathways involved in the intestinal absorption or synthesis of nicotinamide, α-tocopherol and polyamines in naked mole-rats and to consequently reduce their lifespan before a firm cause-effect-relationship between the accumulation of such ‘longevity molecules’ and the phenotype can be established.

Another difficulty inherent to the interpretation of the present results concerns the actual source of the longevity-associated molecules. For example, several molecules that are increased or reduced in naked mole-rats are most likely plant-derived: indole-3-acrylic acid and the saturated fatty acids with uneven-numbered carbon atoms (heptadecanoic and nonadecanoic acid), which all are diminished, and hippuric acid, which is augmented. Hippuric acid levels are known to rise with the consumption of phenolic compounds [42]. Thus, dietary differences in the two species (which do not receive the same chow) may dictate part of the discrepancies in the metabolome. Another molecule that is scarce in naked mole-rats is 3-Indole propionic acid, most likely a bacterial metabolite, contrasting with the fact that a fungal metabolite, azelaic acid, is overabundant. Hence, there may be major differences in the microbiota that explain some of the metabolic discrepancies found between the two species.

It is important to note that the longevity of the mammalian meta-organism composed by the host and is microbial communities is dictated by the systems property of this ecological unit. Thus, both mice and human show age-related shifts in the intestinal microbiota [43–45], and such shifts can actually contribute to the age-associated organismal decline [46], at least in the context of progeroid syndromes [47]. The body content of vitamins from the B series including nicotinamide as well as of polyamines and bile acids are strongly influenced by the intestinal microbiota (because bacteria are able to synthesize them), supporting the intriguing, yet-to-be-confirmed hypothesis that (some of) the longevity-associated metabolic alterations found in naked mole-rats actually reflect a particular gut flora [48]. Thus, one species that is highly abundant in the gut from naked mole-rats, *Bacillus megaterium* [49,50], is also particular efficient in polyamine biosynthesis [51,52]. These results call for an extensive functional characterization of the naked mole-rat microbiota with respect to its possible longevity-conferring properties. The polyamine spermidine is known to induce autophagy in multiple model organisms including mice [53–56], and autophagy induction accounts for its lifespan-extending effects [35–38]. Of note, autophagy reportedly is increased in naked mole-rats [57,58]. Hence, it will be tempting to investigate whether the depletion of polyamine (by inhibition of their biosynthesis by bacterial and host enzymes or by inhibition of their intestinal uptake) would reduce autophagic flux in naked mole-rats, thereby reducing the fitness of this species.

In synthesis, we identified several metabolites that may explain the exceptional longevity of naked mole-rats. Future studies are required to understand the mechanistic bases of their accumulation as well as their actual contribution to the suppression of the aging process.

## MATERIALS AND METHODS

### Animal maintenance and experimental procedure

Animal experimental protocol was approved by the French National Ethical Committee ComEth Anses/ENVA/UPEC (authorization N°12114-2017110916247504-v3). Nineteen male naked mole-rats (1-year-old (n=6), 4 years old (n=6) and 10 years old (n=7)) and twenty-two C57BL6/J male mice (1-1.5 months old (n=7), 6-10 months old (n=10) and 20 months old(n=5)) were used in this study. Naked mole-rats were housed in Plexiglas cages interconnected with tubes to simulate burrows with tunnel systems. Naked mole-rats were kept in the dark, at a temperature of 28-30°C, and 70% humidity.

All animals were fasted overnight and all blood samples were collected in the morning (8-10 am). The blood was collected in heparinized tubes and centrifuged at 3000 rpm, for 10 min at 4°C. Plasma samples (30-50µl) were collected and stored (− 80 °C) until metabolomic analysis.

### Standard and reagents

Acetonitrile (Sigma Aldrich); Isopropanol (Sigma Aldrich); Methanol (Sigma Aldrich); Chloroform (Sigma Aldrich); Acetic acid (Sigma Aldrich); Formic acid (Sigma Aldrich); Methoxyamine hydrochloride (Sigma Aldrich); MSTFA - N-Methyl-N-(trimethylsilyl) trifluoroacetamide (Sigma Aldrich); Pyridine (Sigma Aldrich); 3 nitrophenylhydrazine (Sigma Aldrich); *N*-(3-Dimethylaminopropyl)-*N*′-ethylcarbodiimide; hydrochloride (EDC) (Sigma Aldrich); Sulfosalicylic acid (Sigma Aldrich).

### Sample preparation plasma (lithium heparin)

A volume of 25 µL of plasma were mixed with 250 µL a cold solvent mixture with ISTD (MeOH/Water/Chloroform, 9/1/1, -20°C), into 1.5 mL microtube, vortexed and centrifuged (10 min at 15000 g, 4°C) to obtain protein precipitation. Then upper phase of supernatant was split in three parts: 50 µL were used for GC-MS experiment in injection vial, 30 µL were used for the SCFA (Short Chain Fatty Acids) UHPLC-MS method, and 50 µL were used for others UHPLC-MS experimentations.

GC-MS aliquot was evaporated and 50 µL of methoxyamine (20 mg/mL in pyridine) were added on dried extracts, then stored at room temperature in dark, during 16 hours. The day after, 80 µL of MSTFA was added and final derivatization occurred at 40°C during 30 minutes. Samples were directly injected into GC-MS.

Concerning the UHPLC-MS aliquots (for SCFA method), 15 µl of 200mM 3-NPH and 15 µl of EDC (150mM) were added. The whole was heated at 40°C during 1h. 60 µl of H20 was added and the whole was injected into UHPLC-MS.

Concerning the LC-MS aliquots, the 50 µl collected supernatant was evaporated at 40°C in a pneumatically-assisted concentrator (Techne DB3, *Staffordshire, UK*). The LC-MS dried extracts were solubilized with 150 µL of MilliQ water. Samples were aliquoted for LC methods and backup. Biological samples and QC aliquots are kept at -80°C until injection or transferred in vials for direct analysis by UHPLC/MS.

Concerning the rest of the supernatant and the pellet, 90 µl of methanol with 2% of sulfosalicylic acid (SSA) was added before vortex and centrifugation (10 min at 15000g, 4°C). 130 µl of the supernatant were transferred in a microtube and evaporated. The dried sample were spiked with 100 µl of MilliQ water before injection in UHPLC/MS of the polyamines method.

Aliquots for analysis were transferred in polypropylene vials and injected into UHPLC-MS or kept at -80°C until injection.

### Widely-targeted analysis of intracellular metabolites gas chromatography (GC) coupled to a triple quadrupole (QQQ) mass spectrometer

GC-MS/MS method was performed on a 7890B gas chromatography coupled to a triple quadrupole 7000C (Agilent Technologies, Waldbronn, Germany) equipped with a High sensitivity electronic impact source (EI) operating in positive mode.

Front inlet temperature was 250°C, injection was performed in splitless mode. Transfer line and ion-source temperature were 250°C and 230°C, respectively. Septum purge flow was fixed at 3 mL/min, purge flow to split vent operated at 80 mL/min during 1 min and gas saver mode was set to 15 mL/min after 5 min.

Helium gas flowed through column (J&WScientificHP-5MS, 30m x 0.25 mm, i.d. 0.25 mm, d.f., Agilent Technologies Inc.) at 1 mL/min. Column temperature was held at 60°C for 1 min, then raised to 210°C (10°C/min), followed by a step to 230°C (5°C/min) and reached 325°C (15°C/min), and be hold at this temperature for 5 min.

Collision gas was nitrogen. Scan mode used was MRM for biological samples. Peak detection and integration of analytes were performed using Agilent Mass Hunter quantitative software (B.07.01).

### Targeted analysis of bile acids by ion pairing ultra-high-performance liquid chromatography (UHPLC) coupled to a Triple Quadrupole (QQQ) mass spectrometer

Targeted analysis was performed on a RRLC 1260 system coupled to a Triple Quadrupole 6410 (Agilent Technologies) equipped with an electrospray source operating in positive mode. Gas temperature was set to 325°C with a gas flow of 12 L/min. Capillary voltage was set to 4.5 kV.

10 μl of sample were injected on a Column Poroshell 120 EC-C8 (100 mm x 2.1 mm particle size 2.7 µm) from Agilent technologies, protected by a guard column XDB-C18 (5 mm × 2.1 mm particle size 1.8 μm) and heated at 40°C by a Pelletier oven.

Gradient mobile phase consisted of water with 0.2% of formic acid (A) and acetonitrile/isopropanol (1/1; v/v) (B) freshly made. Flow rate was set to 0.3 mL/min, and gradient as follow: initial condition was 70% phase A and 30% phase B, maintained during 1.5 min. Molecules were then eluted using a gradient from 30% to 60% phase B over 9 min. Column was washed using 98% mobile phase B for 2 minutes and equilibrated using 30% mobile phase B for 2 min. After each injection, needle was washed twice with isopropanol and thrice with water. Autosampler was kept at 4°C.

At the end of batch analysis, column was rinsed with 0.3 mL/min of MilliQ water (phase A) and acetonitrile (phase B) as follow: 10% phase B during 20 minutes, to 90% phase B in 20 minutes, and maintained during 20 minutes before shutdown.

Collision gas was nitrogen. Scan mode used was the MRM for biological samples. Peak detection and integration of the analytes were performed using the Agilent Mass Hunter quantitative software (B.07.01).

### Targeted analysis of polyamines by ion pairing ultra-high performance liquid chromatography (UHPLC) coupled to a Triple Quadrupole (QQQ) mass spectrometer

Targeted analysis was performed on a RRLC 1260 system coupled to a Triple Quadrupole 6410 (Agilent Technologies) equipped with an electrospray source operating in positive mode. The gas temperature was set to 350°C with a gas flow of 12 l/min. The capillary voltage was set to 3.5 kV.

10 μl of sample were injected on a Column Kinetex C18 (150 mm x 2.1 mm particle size 2.6 µm) from Phenomenex, protected by a guard column C18 (5 mm × 2.1 mm) and heated at 40°C by a Pelletier oven. Heat the column more than the room temperature allowed rigorous control of the column temperature.

The gradient mobile phase consisted of water with 0.1% of Heptafluorobutyric acid (HFBA, Sigma-Aldrich) (A) and acetonitrile with 0.1% of HFBA (B) freshly made. The flow rate was set to 0.2 ml/min, and gradient as follows: initial condition was 95% phase A and 5% phase B. Molecules were then eluted using a gradient from 5% to 40% phase B over 10 min. The column was washed using 90% mobile phase B for 2.5 minutes and equilibrated using 5% mobile phase B for 4 min. The autosampler was kept at 4°C.

The collision gas was nitrogen. The scan mode used was the MRM for biological samples. Peak detection and integration of analytes were performed using the Agilent Mass Hunter quantitative software (B.07.01).

### Targeted analysis of Short Chain Fatty Acid by ion pairing ultra-high performance liquid chromatography (UHPLC) coupled to a 6500+ QTRAP mass spectrometer

Targeted analysis was performed on a RRLC 1260 system (Agilent Technologies, Waldbronn, Germany) coupled to a 6500+ QTRAP (Sciex, Darmstadt, Germany) equipped with an electrospray ion source.

The instrument was operated using multiple reaction monitoring (MRM) in negative ion mode with unit resolution for both Q1 and Q3.

The optimized MS/MS conditions were: ion spray source temperature at 450°C, curtain (CUR) gas pressure at 40 psi, gas 1 (GS1) pressure at 30 psi and gas 2 (GS2) pressure at 70 psi. Ion-spray voltage (IS) was set at -4500V, collision-activated dissociation (CAD) at High, entrance potential (EP) at -10V and declustering potential (DP) at -80V.

10 μl of sample were injected on a Column Zorbax Eclipse XBD C18 (100 mm x 2.1 mm particle size 1.8 µm) from Agilent technologies, protected by a guard column XDB-C18 (5 mm × 2.1 mm particle size 1.8 μm) and heated at 50°C by a Pelletier oven.

Gradient mobile phase consisted of water with 0.01% of formic acid (A) and acetonitrile with 0.01% of formic acid (B). Flow rate was set to 0.4 mL/min, and gradient as follow: initial condition was 80% phase A and 20% phase B, maintained during 6 min. Molecules were then eluted using a gradient from 20% to 45% phase B over 7 min. Column was washed using 95% mobile phase B for 5 minutes and equilibrated using 20% mobile phase B for 4 min. The autosampler was kept at 4°C.

At the end of batch analysis, column was rinsed with 0.3 mL/min of MilliQ water (phase A) and acetonitrile (phase B) as follow: 10% phase B during 20 minutes, to 90% phase B in 20 minutes, and maintained during 20 minutes before shutdown.

The software used to operate the mass spectrometer was Analyst (Version 1.7). Peak detection, integration and quantification of the analytes were performed using MultiQuant quantitative software (Version 3.0.3).

### Untargeted analysis of intracellular metabolites by ultra-high performance liquid chromatography (UHPLC) coupled to a Q-Exactive mass spectrometer. Reversed phase acetonitrile method

The profiling experiment was performed with a Dionex Ultimate 3000 UHPLC system (Thermo Scientific) coupled to a Q-Exactive (Thermo Scientific) equipped with an electrospray source operating in both positive and negative mode and full scan mode from 100 to 1200 m/z. The Q-Exactive parameters were: sheath gas flow rate 55 au, auxiliary gas flow rate 15 au, spray voltage 3.3 kV, capillary temperature 300°C, S-Lens RF level 55 V. The mass spectrometer was calibrated with sodium acetate solution dedicated to low mass calibration.

10 μL of sample were injected on a SB-Aq column (100 mm × 2.1 mm particle size 1.8 μm) from Agilent Technologies, protected by a guard column XDB-C18 (5 mm × 2.1 mm particle size 1.8 μm) and heated at 40°C by a Pelletier oven. The gradient mobile phase consists of water with 0.2% of acetic acid (A) and acetonitrile (B). The flow rate was set to 0.3 mL/min. Initial condition is 98% phase A and 2% phase B. Molecules were then eluted using a gradient from 2% to 95% phase B in 22 min. The column was washed using 95% mobile phase B for 2 minutes and equilibrated using 2% mobile phase B for 4 min.

The autosampler was kept at 4°C.

Peak detection and integration were performed using the Thermo Xcalibur quantitative software (3.1.).

### Statistical methods

Raw data were processed and cleaned with R using the GRMeta package (located on Github/kroemerlab). Quality controls consisted in eliminating too low ion signal responses (signal to noise ratio less than 5) and biased metabolites (standard deviation of quality control pooled samples more than 26%). Kruskal-Wallis test with no adjustment were conducted on processed data with R. The heatmap was constructed with log2 normalized data centered around the average abundance from all samples. Metabolites were subjected to hierarchical clustering following the ward. D2 method and using the Euclidean distance. Volcano plots were constructed with a two-sided Wilcoxon to calculate p-values and subtractions of means (on a log2 scale) of the groups (Mice/NMR, and Old/Young).

## Supplementary Material

Supplementary Figures

Supplementary Table 1
