# Supplementary material for: The metabolomic signature of extreme longevity: naked mole rats versus mice
Source: Aging (Albany NY). 2019 Jul 24;11(14):4783–800. doi: 10.18632/aging.102116 (PMC6682510; doi:10.18632/aging.102116)
Supplement: Supplementary Figures [file aging-11-102116-s002.pdf]

SUPPLEMENTARY FIGURES

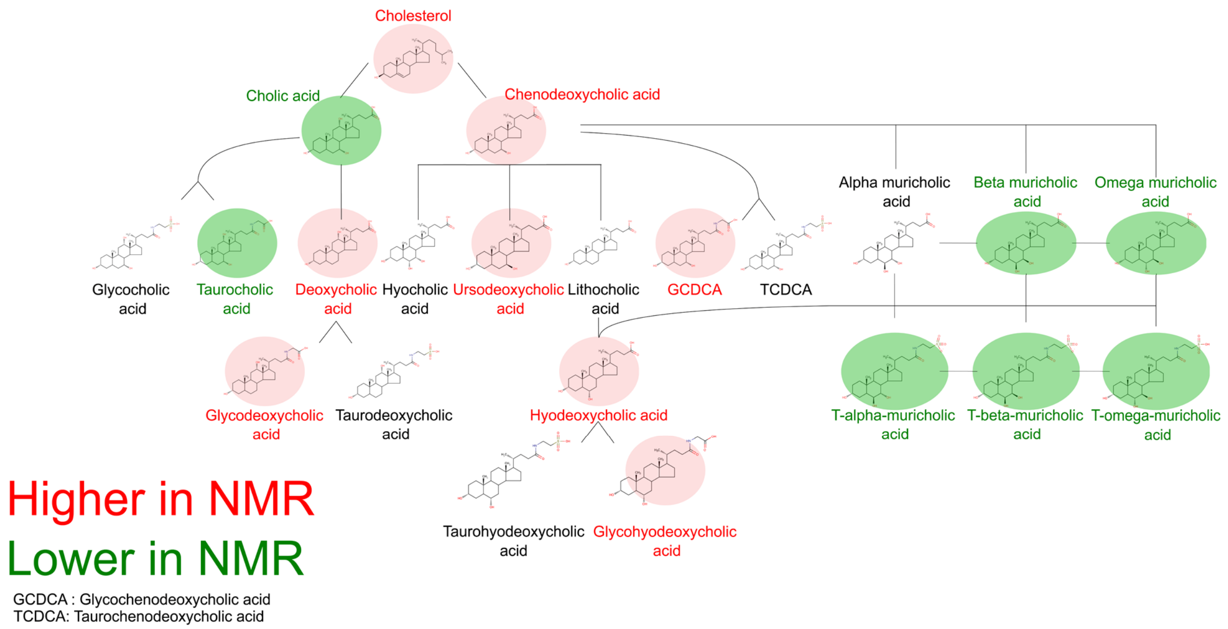

Supplementary Figure 1. Schematic overview of interspecies differences in bile acid metabolism. Note that molecular species that are more abundant in naked mole-rats are marked with pink backgrounds, while molecules that are more abundant in mice are marked in green.

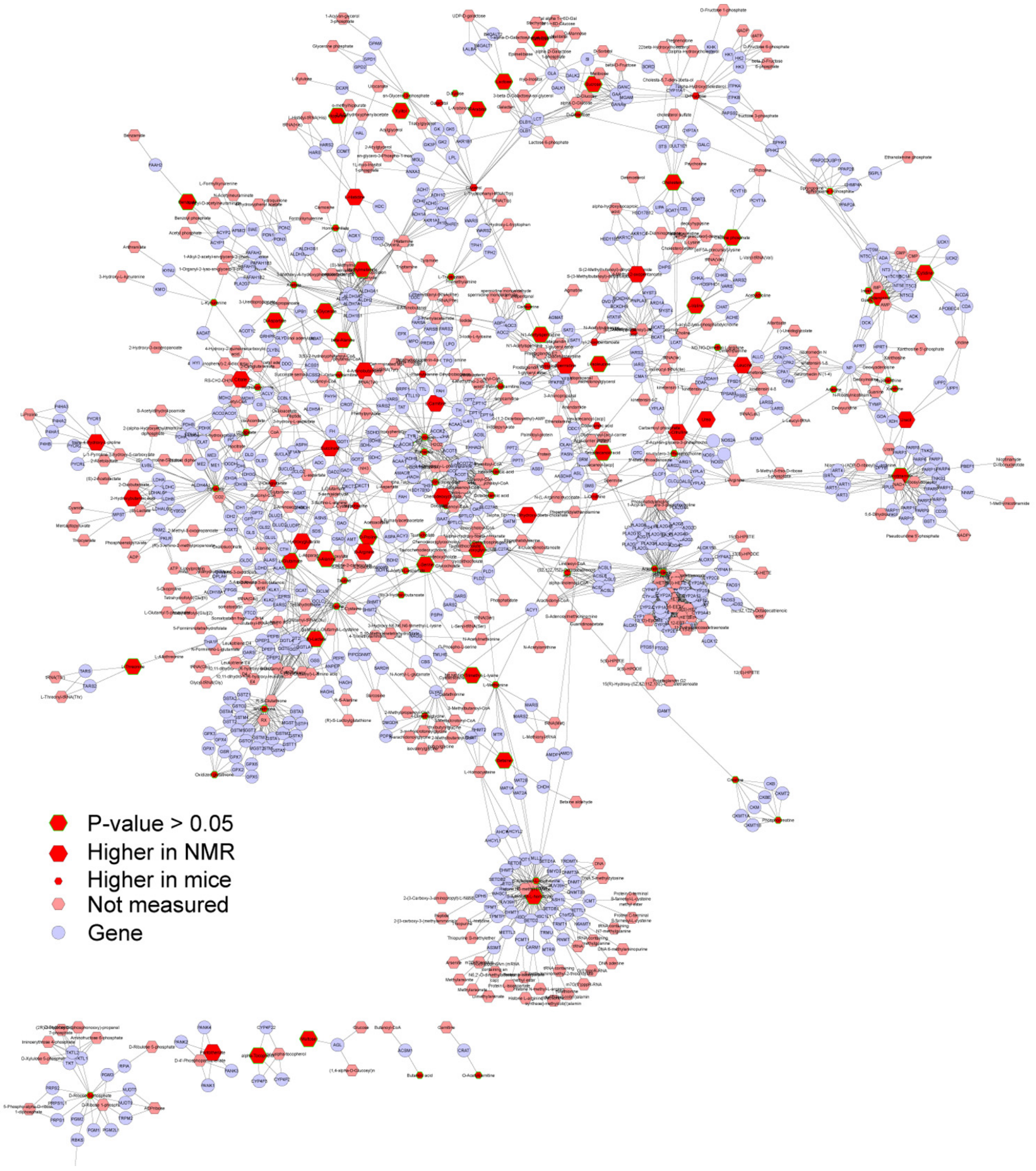

Supplementary Figure 2. Pathway analysis was processed with Metscape app v3.1.3 from Cystoscape software v3.7.1 (ref http://cytoscape.org). Fold-changes were calculated by dividing means of groups. P-values were obtained by means of the two-sided Wilcoxon test.

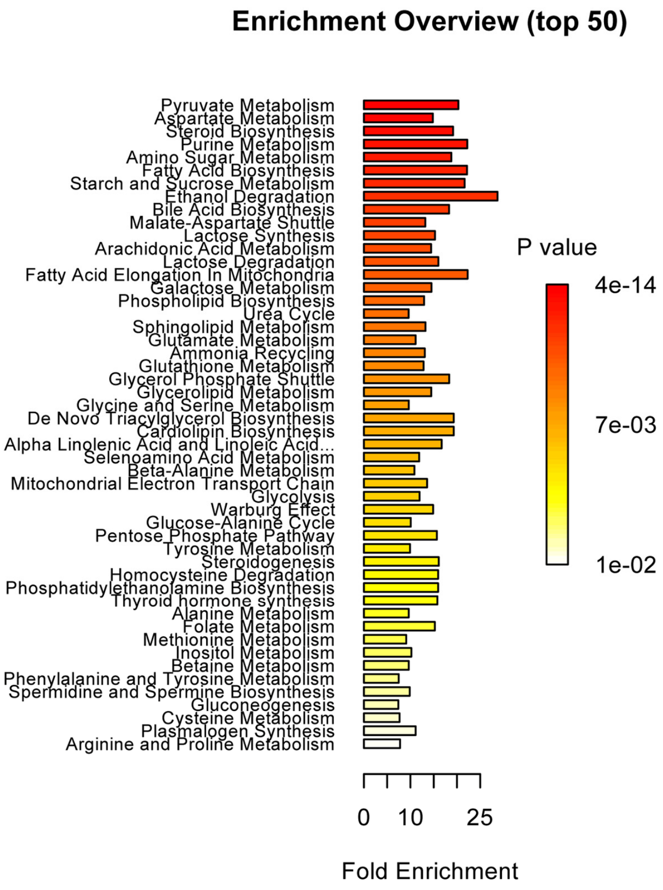

Supplementary Figure 3. Quantitative enrichment analysis was processed with MetaboAnalyst 4.0 (https://www.metaboanalyst.ca/). Data were log-transformed and mean-centered. SMPDB pathway-associated metabolite sets was used, with a threshold of 3 compounds minimum per pathway.
